# Animal Business: an Ethical Exploration of Corporate Responsibility Towards Animals

**DOI:** 10.1007/s41055-021-00094-9

**Published:** 2021-10-30

**Authors:** Monique Janssens

**Affiliations:** Rotterdam School of Management, Erasmus University Rotterdam, Rotterdam, Netherlands

**Keywords:** Corporate responsibility, Animal welfare, Moral status, Business practice, Stakeholders

## Abstract

The aim of this paper is to take normative aspects of animal welfare in corporate practice from a blind spot into the spotlight, and thus connect the fields of business ethics and animal ethics. Using insights from business ethics and animal ethics, it argues that companies have a strong responsibility towards animals. Its rationale is that animals have a moral status, that moral actors have the moral obligation to take the interests of animals into account and thus, that as moral actors, companies should take the interests of animals into account, more specifically their current and future welfare. Based on this corporate responsibility, categories of corporate impact on animals in terms of welfare and longevity are offered, including normative implications for each of them. The article concludes with managerial implications for several business sectors, including the most animal-consuming and animal-welfare-threatening industry: the food sector. Welfare issues are discussed, including the issue of killing for food production.

During the last few decades, societal and philosophical support for the idea that animals should receive more legal and moral protection has grown. Consumers, investors and NGOs have become critical of the treatment of animals by corporations and nations (Amos and Sullivan 2018; Special Eurobarometer 2015). Nevertheless, normative explorations of the responsibility of companies towards animals are scarce (Van Liedekerke and Dubbink 2008).

Therefore, in this paper I will explore the ethical responsibility of businesses towards animals. I argue that animals have a moral status (section 2). In section 3, I discuss how this leads to an obligation of moral agents to consider their interests, and conclude that these are served best by considering their welfare. In section 4, I argue that the moral obligation of individual humans can be expanded to collectives, including companies. In section 5, I explore some aspects of animal welfare in the corporate context, including the issue of ending their lives. In section 6, I draw conclusions and offer managerial implications. I conclude with recommendations for research and several industries.

## The Moral Status of Animals

In this section I explore the concept of moral status and identify how it applies to animals. I look into the concept of moral status in relation to sentience, and identify to which animals it is applicable. I summarize relevant biological knowledge of animal sentience and conclude that some groups of animals can make a moral claim on moral agents, based on their sentience.

### Moral Status and Sentience

‘To have a moral status is to be morally considerable, or to have moral standing. It is to be an entity towards which moral agents have, or can have, moral obligations’, states Warren (1997:3). She explores which entities have a moral status. In that search, she considers the views of several philosophers, such as Taylor on the one hand, who attributes moral status to each goal-oriented organism with a tendency to maintain its existence and reproduce, and Kant on the other hand, who limits moral status to rational moral agents. Warren argues that sentience, or the capacity to feel pleasure or pain, should be the key concept for moral status, because there is a general agreement that it is wrong to inflict pain on those who can experience it. She distinguishes the Sentience Only view (sentience is a necessary and sufficient criterion for moral status) and the Sentience Plus view (sentience is a sufficient, but not necessary criterion for moral status, because there can be other sufficient criteria), and distinguishes as well between *some* moral status and *full* moral status. She argues that there may be valid reasons for ascribing moral status to some non-sentient entities (e.g. biological species or ecosystems) and for ascribing a stronger moral status to some sentient beings than to others. Although this system of moral status is beyond our scope, it is relevant to our case that sentience of organisms provides a moral status, and is therefore a reason not to kill them or cause them pain (Warren 1997).

Several authors agree with Warren on the moral status of sentient animals. Rollin (2016b), for example, agrees with Warren that sentience is a sufficient condition for moral status. He stretches the notion of pleasure and pain to any positive or negative mental state, such as boredom, and prefers to speak of what *matters* to animals. From a Kantian perspective, Korsgaard also agrees to attribute moral status to sentient animals, stating that animals pursue the objects of their inclinations just like humans do, and that both are creatures for whom things can go well or badly (Višak and Garner 2016). Singer’s (1975/2009) utilitarian view on ethical evaluation and decision making is based on a moral status of animals dictated by their sentience. Regan (1983/2004) acknowledges moral status to individuals who have beliefs, desires, preferences, intentionality, memory and a sense of their future. He defines mammals as subjects-of-a-life and therefore ‘moral patients’: they have a life that is theirs and can undergo moral acts from moral agents.

For our purpose, there is no need to explore the differences between the views or levels of moral status. From here I take it as a starting point that *at least some* animals have a moral status. One remaining problem is the difference between sentience as a sufficient condition for moral status, which most authors accept, and the narrower group of animals to which Regan attributes moral status. Using his definition of subjects-of-a-life, he first confined moral status to mammals at least one year old (Regan 1983/2004), and later argued that birds probably should be added, and maybe fish too (1983/2004), which takes the demarcation line closer to the sentience threshold, as we will see later. Despite the remaining fuzzy area, I accept sentience as a sufficient condition for moral status and explore now which groups of animals are considered sentient.

### Demarcating Animals with Sentience

Sentience is the ability to experience positive and negative affective states, such as pain, grief, hunger, thirst, happiness, fear, and pleasure (Duncan 2006). Apart from that, anticipation, memory and coping with challenges play a role (Spruijt et al. 2001). Knowing that a positive or a negative state is to come, as well as remembering it, can intensify the experience (Varner 2012). Varner sees a continuum from mere sentience (of beings who can experience positive and negative mental states) via near-personhood (of beings who have a sense of their mental states in the past and the future) to personhood (of beings who can place their mental states of the past and the future in their life story, which is in line with DeGrazia 2006). Akhtar (2011) and Rollin (2011) on the other hand argue that animals may experience pain more negatively than humans do, because animals have less understanding of the situation. Without drawing conclusions about differences based on memory, anticipation, and understanding, I will stick to the concept of sentience as a sufficient condition for moral status.

Biological research uses four types of evidence for sentience: a nervous system similar to the human nervous system, behaviour in reaction to injury that is similar to human behaviour in reaction to injury (crying, howling, shrieking, moaning, etc.), the presence of sense organs and/or behaviour indicative of perceptual ability (not sufficient for sentience, but apt to be accompanied by it), and the presence of neurochemicals that in humans are related to the experiencing of pleasure, pain, or emotion (Warren 1997). Based on these criteria, researchers have drawn conclusions about several groups of animals, which I have listed in Table 1. All vertebrates – mammals, birds, reptiles, amphibians and fishes – seem to be sentient. There are signs that many other groups of animals may be sentient (e.g. crustaceans) or deserve the benefit of the doubt (e.g. particular insects and molluscs). Therefore, at least vertebrates have a moral status.

**Table 1:** Current knowledge of animal sentience

Animal group*	*What is known of their sentience*
Mammals	Experience pain and pleasure (Bateson 1991; Dawkins 1980)
Birds	Probably experience pain like mammals (Marino 2017; Nasr et al. 2012; Varner 2012)
Reptiles	Probably experience pain like mammals (Stoskopf 1994; Varner 2012)
Amphibians	Probably experience pain like mammals (Stoskopf 1994; Varner 2012)
Fishes	Probably experience pain like mammals (Braithwaite 2010; Bovenkerk and Meijboom 2012; Brown 2015; Chandroo et al. 2004; Elder 2014; Varner 2012)
Cephalopods (squid)	Highly sophisticated and poorly understood nervous system, added to list of sentient animals in European animal experiment legislation (Crook and Walters 2011; Fiorito et al. 2014)
Other molluscs (oysters, mussels, snails, sea slugs etc.)	Motivational states and cognitive capabilities in some species that may be consistent with capacity for states with functional parallels to pain (e.g. avoiding food previously combined with electric shock) (Crook and Walters 2011)
Crustaceans (crabs, lobsters, crayfish, shrimp)	Different systems from those of vertebrates, similar functions, possibly similar experience of suffering (Elwood et al. 2009)
Insects	Reaction to harm and potential harm, no change of activity when bodies are damaged, uncertainty about pain experience, species diversity too large to generalize (De Goede et al. 2013b); pessimistic interpretations and lowered levels of dopamine, octopamine, and serotonin of agitated honeybees (Bateson et al. 2011)

*Grouping of animals not based on taxonomic ranking but on available literature

## Moral Obligations

If entities have a moral status, they can make a moral claim on moral agents (Gruen 2017). Their moral status represents general claims about the ways in which moral agents ought to conduct themselves towards them. It is not necessary for animals themselves to be able to express this moral claim. Infants cannot express any moral claims either, but their moral status is beyond doubt. Acknowledging their moral claims, we make sure they are represented by grown-ups who can express their claims. Still, the claim is not their representatives’, but theirs. The same holds true for sentient animals. Although they are represented by people or NGOs, the moral claim remains theirs.

If vertebrates have a moral status, which we concluded above, it is an obligation of moral agents (in general: humans) to give them moral consideration. What do these obligations consist of? What should be done in the event of conflict of interest? To determine how we should act as humans, we need ethical theories. Different ethical approaches have explored human moral obligations to animals. I will now briefly present the most relevant ones.

### Utilitarianism

The most important philosopher of a utilitarian approach to animal ethics is Singer (1975/2009, 1981/2011). His ground for embracing utilitarianism is the idea that for ethical reasoning one should see one’s own interests as one set of interests among the sets of interests of other beings, who are equally important. Humans, as rational creatures, should acknowledge that it does not matter whether someone in pain is our kin or a stranger, our neighbour or someone far away, black or white, male or female, our own species or another species. We should treat animals according to their species-specific and individual interests. Utilitarianism has strong rational grounds. There is not a lot we can know about ‘the other’ (human or non-human) but what we do know is that positive experiences are better than negative ones. If all sentient beings prefer positive experiences, then we should strive for the most positive experiences for all. Utilitarianism has a strong case in claiming that the interests of sentient animals as *their* interests should be taken into account in assessments of right and wrong.

### Duty-Based Approaches

The most well-known duty-based approach to ethics is Kant’s deontological theory, which acknowledges no direct human duties with regard to animals (Kant 1785/1902/1996; Kant 1963). Kant claims that rationality and autonomy (being able to make choices about one’s own life) are central features for having inherent worth (i.e. being an end in itself), and therefore for obtaining moral consideration. These features of others give us reasons to respect their choices. As animals would not have these features, moral agents have only indirect duties towards them. This would mean that we should consider their interests only for the sake of other moral agents.

This stance had been challenged by several successive duty-based approaches. I mentioned earlier that Regan (1983/2004) offers a duty-based approach to the moral status of animals. He argues that subjects-of-a-life should be treated respectfully and not harmed, which implies direct moral duties of moral agents. According to Regan, this rule should only be overridden if not harming them would harm an even greater number of moral patients (including moral agents, who are also moral patients). Garner (2013) states that at least some animals have an interest in not suffering, which implies that moral agents have duties towards them. Kaldewaij (2013) shows that even starting from the Kantian idea that similarities between rational actors lead to direct duties towards humans, direct duties of humans towards animals are defendable.

### Telos-Based Approaches

A third group of approaches is based on Aristotelian virtue ethics and on *telos*, the purpose by which one flourishes. Nussbaum (2007) argues that, just like humans, animals have a *telos* and should be allowed to exercise certain capabilities so that they can flourish. They should therefore be granted the following: a life (and continuing it, although killing non-sentient animals for good reasons may be acceptable), bodily health, bodily integrity (no violence, abuse, and other forms of harmful treatment), access to sources of pleasure (free movement in an environment that pleases their senses), emotions through attachment to others (feelings of friendship and care, no fear), practical reasoning (to the extent the capacity is present, which differs by species and by individual), affiliation (attachments and bonding with their species), relationships with other species (as preferred), play, and control over their environment (Nussbaum 2007). Her translation of telos into concrete capabilities is extremely useful for case assessments.

Although Rollin (2006) claims to combine approaches in a pragmatic way – because in his view it is obvious that sentient animals’ positive and negative experiences *matter* to them – the core of his approach is also the animal’s *telos*: ‘the unique set of traits and powers that make the animal what it is, the “pigness” of the pig, the “dogness” of the dog’ (Rollin 2016a, 2016b:51), from which needs and desires flow. Not satisfying these needs and desires results in poor welfare, which should be avoided (Rollin 2016a, 2016b).

### Combined Approaches and Other Approaches

Several authors have taken other routes and reached similar conclusions. Cochrane (2013) combines utilitarianism with a duty-based view. He argues that the interests of humans and animals can and should be weighed against one another. Garner (2013) offers a contract approach and finds a thorough obligation of humans towards animals. Donaldson and Kymlicka (2011) see animals as fellow citizens who should be granted rights on grounds of their ability to communicate, work and relate to others. Their approach is viewed as one of the triggers of the political turn in animal ethics, a paradigm shift characterized by, among other things, less focus on ethical theory, more focus on liberal values and positive rights, and a pragmatic attitude towards political engagement and compromise (Milligan 2016). Schmitz (2016) as well pleads for political and institutional change and argues that it cannot be right to maintain institutionalized practices that make animals suffer for trivial human purposes.

### Similarities

I have mentioned several approaches of animal ethics with serious theoretical differences. Nevertheless, almost all of them – except Kant’s theory, but including other duty-based approaches based on and reacting to it – point in the direction of direct human obligations to sentient animals. Although I do not advocate choosing and mixing ethical approaches as it suits my case, I want to remark that some of the approaches lead to similar conclusions about using animals. According to utilitarianism, for example, animals in industries are harmed in such a way that we should stop these practices unless they are necessary for a very crucial human interest. Curing deadly human diseases could be such a cause, but enjoying the taste of meat is not. If this stance were applied as a rule of utilitarianism, it would come close to a duty of humans not to harm animals for unnecessary purposes. Telos-inspired capabilities would also reject the ways in which animals are currently kept and slaughtered for trivial purposes.

I conclude that humans should attribute sentient animals a moral status and consider their interests. Though the exact demarcation line of sentient animals remains undetermined – I will go into that later – the broad spectrum of ethical approaches gives us many reasons to give several animal species the benefit of the doubt (if any) and to avoid harming them.

## The Responsibility of Companies

I have argued that humans should attribute sentient animals a moral status and take their interests into account. Does this mean we can talk of moral responsibilities of companies towards animals? I will now investigate what responsibility is and how individual, collective and corporate responsibility are related.

### Individual versus Collective Responsibility

According to Scanlon (1998/2000), there are two types of responsibility. Attributive responsibility means that an action can be attributed to an actor who is properly subject to praise or blame. Substantial responsibility means that an actor’s specific role or position requires them to act in a certain way. If this type of responsibility applied to companies, it would mean they owe something to someone, for example to society, to consumers, or to animals. However, companies are not persons, so in their case responsibility could work out differently. Therefore, I will first explore the relationship between responsibilities of persons and those of companies.

Pettit (2007) offers three necessary conditions for fitness to being held responsible: there is an autonomous agent facing a value-relevant choice involving the possibility of doing good or bad, or right or wrong; the agent can judge the value of the options (understands it and has access to supportive evidence); and the agent has the control that is necessary for being able to choose. Members of groups can be held responsible for their part in the design of the group, for the actions of the group (as members, unless they protested) and for acting in the group’s plans. These conditions often apply to decision-makers in companies, who according to Isaacs (2011) bear more responsibility for the consequences of their actions than individuals outside companies, because of their powerful position. Isaacs explores the issue that collective moral responsibility requires collective intentions, whereas collectives cannot have intentions, which are mental states. Her argument is that individual intentions can set in motion collective actions, and therefore individuals are responsible for the consequences of their actions within a collective. At the same time, they can be bound by formal policies and structures, such as interests, attitudes, practices and cultures. I assume with Isaacs that individual agency is not absorbed by collective agency, but, on the contrary adds power.

In addition, Isaacs states that when a collective has obligations that are not met, this failure shapes and alters the obligations of individuals as members of the collective. This means that powerful people in companies can, and should, take individual responsibility and use their power to initiate responsible collective corporate actions. Groups of these powerful individuals (boards, for example) are collective agents. Apart from these existing collective agents, there are, according to Isaacs, potential collective agents: groups that could be formed to act upon certain moral issues. That these new collectives can have a positive influence on a responsible attitude of companies regarding animals is confirmed by Janssens and Van Wesel (2018). But, Isaacs warns, fuzziness of roles and tasks can pose a challenge to the outcomes. Therefore, against the background of collective responsibility, members are morally required to sort out their roles and tasks, so that collective actions can take place. The individual is not responsible for change, but for doing her part. This also holds true for issues that were not caused by the collective, and of which the cause may be unclear. Vagueness about what can be done, or a wrongful social practice combined with widespread ignorance about the wrongness or harmfulness of actions, diminishes the individual’s responsibility.

### Corporate Responsibility

Companies have societal responsibilities, as has been argued thoroughly from the point of view of both business ethics (Kaptein and Wempe 2002, Kolstad 2007) and CSR (Van Marrewijk 2003; Visser 2014). These responsibilities have hardly been applied structurally to the interests of animals (Janssens and Kaptein 2016; Van Liedekerke and Dubbink 2008). Arguing from the idea that individuals in collectives, including companies, bear responsibility for the consequences of their actions, there are two options: the functional model (the company cannot be perceived as an autonomous moral agent) or the autonomy model (the company can be perceived as an independent social entity that can be held responsible for the effects of its actions) (Kaptein and Wempe 2002). I think the autonomy model does the most justice to the fact that companies make policy choices through institutional decision-making processes that can be distinguished from individual staff choices and that are embedded in corporate practices. Therefore, I will proceed from the autonomy model.

Carroll (1991) distinguishes four categories of responsibilities of companies, which together constitute CSR. The basis of the pyramid of corporate responsibilities consists of economic ones: making a profit for investors and creating jobs. Next come legal responsibilities, then ethical ones, and finally, on the top of the pyramid, philanthropic responsibilities. Though responsibilities towards animals could appear in each of these categories, I will focus on ethical responsibilities: those which are not prescribed by law but are more binding than philanthropy – the responsibility to do what is right, just and fair, and to avoid harm.

Returning with this definition to Scanlon (1998/2000), we discover that both attributive and substantial responsibility can occur in companies. A retailer who uncritically continues to sell meat from a producer who has been found guilty of animal abuse bears attributive responsibility: There is a direct relationship between not acting and the consequences, for which the non-actor can be blamed. At the same time, the retailer bears a substantial (role-based) responsibility to monitor the interests of animals through the production chain. For the scope of this chapter it makes no difference in which cases corporate responsibility is attributive or substantial. They may supplement and intermingle.

### Animals as Stakeholders

Stakeholder theory may offer supporting arguments for corporate responsibilities towards animals, because it states that companies have an ethical responsibility to create value for stakeholders (Freeman et al. 2010; Freeman and Velamuri 2008). A broadly accepted definition of a stakeholder is: any group or individual who can affect or is affected by the achievement of the organization’s objectives (Donaldson and Preston 1995, Freeman et al. 2010). Animals differ from (other) stakeholders in two possibly relevant ways.

The first difference that may be relevant is that they are non-human, but the definition is unclear about whether being human is a necessary feature of stakeholders. The definition does say, however, that it is about groups or individuals who can affect or are affected by the achievement of the organization’s objectives, which can be the case for animals. The three animal ethics approaches I discussed above agree that sentient animals have interests. Therefore, there is undeniably something at stake for them when business activities have an impact on their lives, which makes them potential stakeholders. This stance is supported by Hart and Sharma (2004), who argue in favour of including currently marginalised stakeholders like the poor, the weak, and the non-human, and by Mitchell et al. (1997), who classify animals as ‘dependent stakeholders’: those who lack power but who have urgent legitimate claims as ‘dependent’, ‘because they depend upon others for the power necessary to carry out their will’ (p. 977).

This classification of being dependent of others to look after your interests and speak up for you leads us to the second difference: that animals in cannot speak for themselves (at least not in human language). This can be overcome by letting animal-protection NGOs speak for animals (Carroll 1991), using scientific knowledge about their preferences (Janssens and Van Wesel 2018), observing their behaviour (Wemelsfelder et al. 2000) and putting an effort in communicating with them (Meijer 2017). Webster (2006) describes farm animals as stakeholders of farming, besides farmers and customers.

An important critic of stakeholder theory is Heath (2014), who argues that a robust moral code can be derived from the fundamental obligation of a manager to maximize shareholder value within the framework of the law. His main argument is that this is the best way to make managers run the firm in a way that is in the long-term interest of those with a stake in the firm, especially in terms of continuation of business operations. As we have seen earlier, stakeholders are those who are affected by the company’s actions, those for whom there is something at stake, which can be the case in different ways. For some who are affected, a different type of doing business, or even stopping operations, may be in their interest, while for others the solution may lie in changing to innovative products or processes that are friendlier to them but are nevertheless profitable and sustainable. It is this type of sustainable continuation that may be overlooked by shareholders but could in the end be in the interest of all affected.

The conclusion should be that powerful people in companies bear an individual responsibility for the impact of their actions on the interests of animals. In addition, the company has an institutional responsibility. Both levels of responsibility may be attributive or substantial, based on CSR or stakeholder theory. It makes no decisive difference. From here on I will therefore speak of corporate responsibility, which can be fulfilled by actions of individuals within the company or of the company as an institution.

### The Extent of Corporate Responsibility

Animals can be treated badly by companies (Engster 2006; Fraser 2008), or even be abused on a large scale (Braithwaite 2010; Foer 2009; Leder 2012; Singer 1975/2009). The extent of professional and corporate responsibility may differ by context. A remote farmer in Sudan who is not aware of the increasing academic recognition of moral obligations towards animals cannot be expected to proactively transcend local norms for treating animals. Multinationals, by contrast, operating on a global playing field, can be expected be aware of changing public norms and increasing academic knowledge, and to act accordingly. They have both the resources to conduct thorough research before they make policy decisions (Isaacs 2011; Pettit 2007) and the opportunity to cooperate with researchers (Janssens and Van Wesel 2018). The responsibility of a company who produces and sells locally may be restricted to its own actions, whereas the responsibility of a company in the middle of an industrial network and an extended production chain may be much broader. Chain responsibility, for example, is confirmed by Eriksson and Svensson (2016).

A limitation to responsibility of individual actors in a company is that one can only be held responsible if one has the control necessary to make and implement choices (Pettit 2007). This restriction matches Isaacs’ (2011) statement that sometimes an individual or an inter- or intra-corporate group may not have the power to make changes. In that case, Isaacs adds, the company should help these individuals or groups to design their roles in such a way that power is added. If the company itself lacks power, it can search for ways of extending their power to make changes (Janssens and Van Wesel 2018).

## Animal Welfare

Having argued that companies have a responsibility to take the interests of animals into account, I will now consider what the interests of animals consist of. If humans have moral obligations towards animals, this means that they have an obligation to consider the interests of animals in their actions, or the animals’ ‘good’. Sumner (1996:35-37) chooses not to talk of interests when talking about the subjective ‘good’ of beings, but of welfare, because the term interests is too ambiguous: “on the one hand my interests are the same as my concerns (what I am interested in), while on the other my interest (self-interest) is the same as my welfare.” For the purpose of this study, it is functional to embrace this choice en reflect on it in the given context, in the first place because animal welfare is a common term in both societal and academic practice, secondly because it is the central value that should be maximised in utilitarianism (Singer 1975/2009, 1981/2011), and thirdly because translated into practice it gets close to the notion of not being harmed of Regan (1983/2004) and part of Nussbaum’s list of capabilities that animals should be allowed for being able to live according to their telos (e.g. bodily health, access to sources of pleasure, relationships with other species, play, control over their environment) (Nussbaum 2007). I will now say a bit more about the meaning of the term welfare in this context, without trying to offer a full operationalisation of what animal welfare beholds.

### The Meaning of the Term Animal Welfare in this Context

Sumner (1996) defines welfare as authentic (not ‘fake’), subjective (valuable to me) happiness. In his view it is more than a mental state: welfare is about the relationship between the subject and the world and has to do with overseeing options and deciding in a way that is true to one’s goals. This may be true for humans, but does it apply to animals as well? Can animals be ‘tricked’ into non-authentic or non-subjective ‘preferences’? To check this, we imagine a hungry sow, lured away from her piglets with food and then separated from them permanently. Her fake preference will be to take the food, but her authentic preference would probably have been to go on taking care of her litter and feed herself later, had she been able to oversee the consequences of her options. Therefore, authentic preference seems to be applicable. Secondly, can animals have non-subjective preferences? A subjective preference of a dog could be to curl up with one of its owner’s old sweaters, which will make her feel happy. The same sweater has no value to the neighbour’s dog, who doesn’t like the smell. Snuggling this specific sweater is not a preference of the neighbour’s dog, although someone might conclude from the first dog’s reaction that ‘dogs like this sweater’. This means that the notion of subjective preferences can apply to animals as well. Therefore, the definition of animal welfare we will keep in mind is: the fulfilment of an animal’s authentic, subjective preferences, those being the preferences this specific animal would have, overseeing and understanding the consequences of relevant options.

There is another aspect to animal welfare that is relevant to this argument. That is the awareness that it is impossible to identify when an individual has an acceptable level of welfare. Welfare takes a continuum from extremely negative to extremely positive. Which level of welfare is acceptable and how it can be measured and weighed against other interest is hard to determine and influenced by context (Ohl and Van der Staay 2012).

### The Harm of Taking Life

An ongoing discussion in animal ethics is the question of whether taking the life of healthy animals without causing them pain or distress constitutes harm. Regan (1983/2004) is rather clear about this: moral patients have an inherent value, and therefore killing them is a violation of their moral rights. It is only allowed under specific threatening circumstances. The telos-based approaches are also clear on the issue: Nussbaum (2007) includes continuing life in the list of capabilities we should allow sentient animals, and therefore taking their lives is harming them; Rollin (2006) takes the position that taking the life of an animal needs thorough justification, which he thinks is lacking in, for example, the case of eating them.

At first glance one would think that from a utilitarian perspective, taking life painlessly is not diminishing welfare as long as the animal is not aware of death approaching. No harm has been done before death, and from the moment of death, there is no living animal anymore to have an interest in welfare, and therefore no welfare of an animal is taken away (Broom 2011; Webster 1994). This view is opposed by those who claim that cutting an animal’s life short is taking away its future welfare, and therefore is wrong (Balcombe 2009; Bovenkerk and Braithwaite 2016; Deckers 2016; Kagan 2016; Kasperbauer and Sandøe 2016; Singer 1981/2011; Višak 2015; Yeates 2010). For preference-utilitarians, fulfilment of preferences is the central value that should be maximized (Singer 1993/1999). In this view, killing is taking away future fulfilment of preferences, which makes the loss and therefore the harm greater for those with future-oriented preferences (Chappell 2013). Though this position has been questioned (Kasperbauer and Sandøe 2016; Yeates 2010), it could be an argument for differentiating ending human lives and ending animal lives, since humans have the ability to plan ahead and look forward to future experiences. Nevertheless, at least some animal species can have a concept of the future that is relevant to the moral significance of continuing life (Bradley 2016; Clayton et al. 2003; DeGrazia 1996). We can imagine very well that for a pig, expectations (after feeding time there will be sun in the back of the meadow) and plans (I will go and lie there then) can be thwarted if it is slaughtered, which results in a loss of the (late) pig’s welfare. At first glance, the pig’s loss appears small compared to the loss of a human planning to become a nurse whose life is cut short. Still, to the pig, living in a shorter time frame, the modest afternoon plan could be half its world. That killing animals prematurely is a harm done to them is confirmed by Bruijnis et al. (2016). My conclusion is that killing is a harm if done to a being that at the moment of death has an interest in fulfilling future preferences and enjoying welfare in the future.

What about killing animals living unpleasant lives or facing an unpleasant future? Would it not be better for them to kill them painlessly? If they are already alive and their situation cannot be changed, this would indeed be the best option for them, but it would be even better to change their situation to a positive one and to stop bringing animals destined to lead lives of misery into the world.

### Replaceability Argument

Expecting that my argument may put moral limits on the use of animals in industries, I want to reflect shortly on the replaceability argument, which could be brought forward against our case. In a nutshell, the argument, employed especially against the utilitarian approach of animal ethics, goes like this: Moral actors have a moral duty to maximize overall welfare, but it does not seem to make any difference to which individual this welfare is attached, except that all individuals count equally. This means that taking an animal’s life is permissible if the animal is replaced by a newly bred animal with at least the same welfare level (Singer 1993/1999). Would it then be ethically neutral to kill animals and replace them with equally happy animals?

One of the answers to this question is from Chappell (2013). He argues that an agent with enough life-saving anti-venom for one person would find it horrible to choose which of two poisoned persons to save. Finding this choice horrible is what makes her a moral agent, and it shows that she accords separate value to the interest of each individual, and that those who count are not mere receptacles of welfare. Their interests count equally but as distinctive intrinsic goods. Trade-offs can be made, but a benefit to person A does not *absorb* a lesser loss by person B. I think Chappell’s argument is well grounded, and applies to animals’ welfare as well, because animals are individuals too (Braithwaite 2010; DeGrazia 2006; Rollin 2011). A similar analysis of the replaceability argument is offered by Višak (2011), who claims that the existence of an animal and the non-existence of a potential animal are incommensurable.

Without analysing the issue of the replaceability argument any further, I will stick to my assumption that killing an animal is an infringement upon the animal’s welfare and therefore a harm done to the animal, even if the animal is replaced. If its life is bad, the effort should go into improving it.

### Categories of Impact on the Welfare of Animals

Different types of impact on animals may lead to different ways of dealing with corporate responsibility for the animals involved. In the first place, it matters whether or not the animals are killed. Other prima facie relevant differences are whether they are living in a positive or a negative state, and, thirdly, whether they are living freely or are kept by the company (or by another company in the chain). Those who keep animals for economic reasons might argue that animals are better off in their care than in the wild. They are indeed protected from many threats, such as unhealthy weather conditions, hunger, and predation (Rollin 2016a, 2016b). On the other hand, in industrial farming many aspects of animal flourishing are ignored or actively suppressed, for example mobility, relationships, interaction with the natural world, and autonomy (McMullen 2015). Degrazia (2011) states that confinement as applied in factory farming and traditional zoos is a wrong done to animals. It is my view that if the animal’s autonomous, informed preference is the safety of the barn, this safety should include a fitting welfare standard.

I will now offer a categorisation of different types if corporate impact on the welfare of animals, based on the above characteristics and existing practices, and formulate corresponding responsibilities they should consider based on my earlier argument that the welfare of animals should be taken into account and that taking their lives counts as taking part of their welfare. For the sake of clarity, I offer pronounced examples for each category, assuming that the negative and respectively positive states are the true states of the animals involved, thereby avoiding the discussion about how happy or unhappy for example a free-range dairy cow is (Višak 2015; Webster 2013).

- *Animals kept in a negative welfare state, bred and killed for economic reasons* (e.g. industrially kept pigs, broiler chickens, minks, some laboratory animals). Companies who bear direct or chain responsibility for them should consider their living and dying conditions critically, look for ways to solve welfare issues, and consider innovating towards alternate products that make killing animals unnecessary.
- *Animals kept in a positive welfare state, bred and killed for economic reasons* (e.g. free-range pigs and broilers). Companies who bear direct or chain responsibility for them should consider their dying conditions critically, look for ways to solve welfare issues, and consider innovating towards alternate products that make killing animals unnecessary.
- *Animals kept in a negative welfare state, used for economic purposes over a longer period* (e.g. industrial dairy cows and laying hens, circus animals of wild species). Companies who bear direct or chain responsibility for them should consider their living and dying conditions critically and consider innovating towards alternate products that make keeping them unnecessary.
- *Animals kept in a positive welfare state, used for economic purposes over a longer period* (e.g. free-range dairy cows and laying hens, roaming sheep). Companies who bear direct or chain responsibility for them should monitor their welfare to make sure there is no decline, consider their dying conditions critically, and, in case of persistent welfare and killing issues, consider innovating towards alternate products that make keeping them unnecessary.
- *Animals as by-products, killed at an extremely young age for economic reasons* (e.g. male chicks and steers). Companies who bear direct or chain responsibility for them should consider their living and dying conditions critically, take measures to solve welfare issues, and consider innovating towards alternate products or production methods that prevent their coming to life as by-products.
- *Free-living animals, killed or harmed for economic reasons or as a result of economic activity* (e.g. fishes, house mice in buildings, frogs on building sites). Companies who bear direct or chain responsibility for them should consider their impact on these animals critically, and look for measures, alternate processes or products that solve welfare and killing issues.

Critics may argue that the categories are neither complete nor absolute. Intermediate categories can occur (animals taken from the wild to be kept), or animals may change categories (sheep living in a positive state shifting to a negative state when transported for slaughter). In this case, in-between categories or shifts can easily be derived.

## Implications and Recommendations

I have argued that companies bear responsibilities towards sentient animals, which oblige them to consider how to prevent inflicting harm on them, including killing, and to protect their welfare. A paradigm shift in companies is necessary to accept that sentient animals have interests that count ethically amongst other interests in and outside the company. Harming animals is ethically negative, and asks for thorough ethical accountability. It cannot be denied that the impact on animals of many industries is far from neutral and should be reconsidered and set against the societal necessity or value of the products and services produced.

We have also seen that implications and considerations may differ by context and impact category. Responsibilities can be limited by vagueness about what can be done, secondly by wrongful social practice combined with widespread ignorance, and thirdly by lack of power (Isaacs 2011). For most companies, operating in contexts of abundant information and shrinking global distances, the first two limiting factors will play a minor role. The third limitation, lack of power, can at least partly be solved by efforts to cooperate within industries and production chains (Janssens and Van Wesel 2018). I have categorised different types of impact of companies on animals and listed appropriate responsibilities for each of them. I will now offer some managerial implications and some recommendations with accompanying informative (not argumentative) references.

### Managerial Implications

Managers of companies who acknowledge their responsibility for those animals that are affected by corporate activity should assess their impact on animal welfare and take steps, based on the above categorization. Although it is not the purpose of this paper to offer an operationalisation of how exactly animal welfare can and should be supported, I will offer a few possible directions of managerial action. For livestock issues, some companies are using the Five Freedoms concept (Janssens and Kaptein 2016). The original Five Freedoms are: freedom from hunger or thirst, freedom from discomfort, freedom from pain, injury or disease, freedom to express normal behaviour, and freedom from fear and distress (Brambell 1965). They were updated several times (Botreau et al. 2007; Brando 2016; Farm Animal Council 2009; Sandøe and Jensen 2011) and were recently expanded to 14 welfare criteria (Brando and Buchanan-Smith 2018), which offer an up-to-date guideline. Another concrete road to taking responsibility for animals in economic processes is to offer them ‘labour rights’ to representation by a union, rest and leisure, and retirement (Cochrane 2016). Although this may be a large step for some companies, looking at animals through this lens may trigger a mind shift. Thirdly, treating animals as stakeholders, and discussing their alleged preferences with NGOs and animal behaviour scholars, can be a fruitful approach.

There are no rational arguments for rejecting responsibilities for reasons of burden. Ethical requirements can be tough, especially if they have remained unrecognized by common practice for a long time. Nevertheless, some weighing of interests is inevitable, for example between animal welfare and protection of the environment. This can be difficult, since it can involve many uncertainties (Janssens and Van Wesel 2018). For a correct weighing process, an ethical assessment can be useful. There are several tools for ethical assessment, like the Ethical Matrix (Mepham 2016), which refines common-sense ethics and facilitates discussion and assessment. Bovenkerk and Meijboom (2012) offer a model for defining the moral status of the animals involved (fishes, in their case) and weighing interests.

Implementation barriers can be overcome by taking leadership, working in partnerships with e.g. chain partners and NGOs, and by celebrating ‘championships’, like targets that have been achieved (Baur and Palazzo 2011; Janssens and Van Wesel 2018; Varner 2012). There is a special role for the CSR manager, who can have a positive influence by communicating and facilitating communication with other parties (Janssens and Van Wesel 2019). Gjerris et al. (2011) propose to introduce four virtues in the relationship between consumers and producers of animal-based products: attentiveness, responsibility, competence and responsiveness. Rollin (1995) sees four problematic beliefs in agricultural communities that stand in the way of improvement: the idea that paying attention to animal welfare is opening doors to animal rights (which many people consider a bridge too far), the conviction that one can talk of animal welfare in a value-free context, the idea that science and ethics are separate worlds, and the notion that research into animal welfare cannot adequately address the animal’s experience of pain. It is a challenge for companies to contribute to eradicating those barriers.

### Recommendations for Food Production and Retail

The most animal-consuming and animal-welfare-threatening industry is probably the animal-based food industry (Francione 2010; Rollin 2006). It has many branches, each with its own issues. I have argued that killing animals is ethically dubious. At the same time, demanding an immediate termination of the killing of animals for food is too remote from practice. I do agree with most of the previously mentioned ethicists that in an ideal world, humans neither eat animals nor use products for which animals are killed as a by-product, and that phasing out is feasible. Nevertheless, I will now offer some information on welfare issues that can lead the way to first, more realistic steps in the right direction.

Aside from death, welfare problems in industrial farming are overwhelming, the main ones being lameness, stereotype behaviour, tail biting or tail docking of pigs, feather pecking or beak trimming of poultry, and exhaustion (De Goede et al. 2013a; Webster 2013). Other examples from Deckers (2016) are: castrating and filing teeth of piglets without anaesthesia, chaining sows to the floor, wearing down cows, separating cows and calves, suffocating and crushing of fish on fishing vessels, inadequate temperatures and rough handling in fish farming, and for several species, long transports under extreme temperatures.

Measures for enhancing animal welfare will differ between groups of animals. Examples are: cage enrichment, more space, better housing, free-range options, social contacts, safer transport, and humane slaughter methods. Webster (2013) introduces the Planet Husbandry concept, which means that animals can live in accordance with their natural needs and are cared for responsibly. Rollin (1995) as well sees options for paying more attention to sources of animal suffering. One way is to engage in research into alternative practices, because industrial killing can almost never be done without pain and stress (Pachirat 2011; Rollin 2006). Breeding dual-purpose animals and introducing new methods for sex determination of eggs, for example, can prevent the early killing of male by-products of egg and dairy farming. Fish industries, for whom the discussion about welfare is extra complex and plural (Bovenkerk and Meijboom 2012), should consult biologists, physiologists, and ethologists to take steps (Bovenkerk and Meijboom 2013). The next ethical challenge is the up-and-coming insect industry (De Goede et al. 2013b), which is popular for reasons of efficiency, low emissions and biodiversity (less pressure on vulnerable fish species). Lacking any other norm, De Goede et al. apply the Five Freedoms, designed for more conventional animal husbandry, to insects, which is a delicate practice. They conclude that there is a need for transparency in the sector and research into the welfare of insects.

If companies would attribute the interests of animals a relatively heavy weight in their ethical assessments, the far-stretching ideal of some, a vegan society, seems feasible in the future. To reach that, companies could invest in the development of imitation meat and cultured meat, and promote vegetables, beans, nuts, and plant-based ‘dairy’ (Deckers 2016). Francione (2010) as well argues in favour of stopping animal use for food (or any purpose), which is possible if it is phased out gradually (Simmons 2016). Still, the road to a vegan society is challenged by the debate about animal casualties from arable farming (e.g. machine kill and pest control) (Davis 2008). Varner (2012) tries to solve that problem through innovations such as nest protection and new methods for expelling animals. Davis also mentions physical, economic, political, religious, historical, legal, psychological and cultural obstacles. Although these are many, they should not prevent companies from doing the right thing.

### Recommendations for Other Industries

I will now offer some prima facie recommendations for other industries, based on the former argumentation that necessity of products and services and the value they ad for society should be set against the interests of the animals involved.

Fur and leather are controversial because they are luxury products with many alternatives (imitation fur, plastics, cork, pineapple waste). Sheep, goats, llamas, alpacas, angora rabbits, and angora goats are used for wool production, which raises several welfare issues. Angora rabbit wool, for example, is harvested in a painful way. In the sheep industry mulesing, the cutting away of skin to prevent parasite flies from nestling, is problematic. Rough handling can be a problem for all the species mentioned. However, animal-friendly wool production can be morally acceptable if the welfare of the animal involved is taken into guaranteed (Garner 2013).

The major problems in entertainment using animals are confinement, boredom, and cruel training methods. In zoos, deprivation of space, activities and free choice of company can be problematic. Solutions are: substitution of activities (natural ones for new ones) and other forms of habitat enrichment (Keulartz 2016). As a minimum, the animals’ basic needs should be met (DeGrazia 2011). In some cases, animal-friendly training methods can be helpful (Haraway 2008; Hearne 1992). Additionally, one could argue that displaying animals in circuses, zoos and aquariums is not necessary, and that the sacrifice asked from animals for entertaining humans is too high a price. Each company involved should make their own assessment, weighing the ‘good’ of entertainment, and sometimes species conservation, against the interests of the animals involved.

Many industries, including food and pharmaceuticals, use sentient animals in experiments. In most countries the weighing of their interests against those of society is imposed by law and is being done relatively carefully. Other industries could learn from the ethical assessment done by Animal Experiment Committees. Still, ethical issues of animal research deserve attention (Linzey and Linzey 2018).

Even in industries with no direct animal use, for example the raw materials industry, an assessment of impact on animals is useful. Issues that can be encountered here include pest control, pollution, building activities, transport and catering (Janssens and Kaptein 2016; Varner 2012).

## Conclusions

In this paper I have argued that animals count morally, and that humans as well as their institutions, including companies, have moral obligations towards sentient animals, which had led us to the argument that companies bear responsibilities for the animals they have an impact on. Companies should take the welfare and life of animals into account in their ethical assessments and (at least) diminish animal suffering. I think my argument supports the position that companies should assess their current and future impact on the welfare of animals, explore cutting-edge knowledge about the needs and preferences of the animals involved, and draw conclusions on actions to take. The outcome of these assessments and the options for change may differ per context (corporate impact on animals, necessity of the products or services, status quo of the industry, national or international level on which the company operates, influence of the company, et cetera), but it may be clear that some reflection of each company is desirable.

A limitation of this ethical exploration is that many questions remain unanswered. Apart from uncertainty about the precise extent of corporate responsibility towards animals, there is uncertainty about how to weigh conflicting interest of stakeholders (ethical decision models offering only part of the solution), how to deal with interaction with government, and how to assess consumer responsibility in relation to corporate responsibility. Although I limit myself to describing *corporate* responsibilities towards animals, which means excluding the discussion on responsibilities of other parties, such as consumers, NGOs and governmental organisations, one could argue that these parties are relevant for companies in an indirect way. If companies have a responsibility to take the interest of animal into account when making business decisions, this responsibility can be extended to helping consumers, NGOs and governmental organisations to make better choices for animals. This could mean for example: offering non-animal product options; lowering the threshold to buy these products by advertising, discounts and shelve positioning; collaborating with NGOs on animal welfare purposes; and collaborating in governmental animal welfare projects (Janssens and Van Wesel 2018). Kaiser et al. (2021) describe how the COVID-19 crisis offers opportunities for rethinking the food system and its value priorities bottom-up, including the market as part of this “bottom” and addressing animal and human welfare as obvious ethical issues. Another opportunity for this broader-than-corporate approach lies in adding animal welfare explicitly tot the United Nations Sustainable Development Goals (Keeling et al. 2019). I recommend the above topics for further research. It would be useful as well if scholars further explored the implications for different industries and the moral status of several non-vertebrate animal species.

The claim I put on companies may be an unwelcome message to some involved. Nevertheless, change is morally desirable and even obligatory, as it is the only way to end systematic abuse of animals in industries, of which I have argued that many aspects are wrongful. I therefore hope that decision makers in companies will initiate the recommended changes.

## References

[CR1] Akhtar S, Beauchamp TL, Frey RG (2011). Animal pain and welfare: Can pain sometimes be worse for them than for us?. The Oxford handbook of animal ethics.

[CR2] Amos N, Sullivan R (2018). The business benchmark on farm animal welfare; 2017 report.

[CR3] Balcombe J (2009). Animal pleasure and its moral significance. Applied Animal Behaviour Science.

[CR4] Bateson P (1991). Assessment of pain in animals. Animal Behaviour.

[CR5] Bateson M, Desire S, Gartside S, Wright G (2011). Agitated honeybees exhibit pessimistic cognitive biases. Current Biology.

[CR6] Baur D, Palazzo G (2011). The moral legitimacy of NGO’s as Partners of Corporations. Business Ethics Quarterly.

[CR7] Botreau L, Veissier I, Butterworth A, Bracke MBM, Keeling L (2007). Definition of criteria for overall assessment of animal welfare. Animal Welfare.

[CR8] Bovenkerk B, Braithwaite VA, Meijboom FLB, Stassen EN (2016). Beneath the surface: Killing of fish as a moral problem. The end of animal life: A start for ethical debate; ethical and societal considerations on killing animals.

[CR9] Bovenkerk B, Meijboom FLB (2012). The moral status of fish. The Importance and Limitations of a Fundamental Discussion for Practical Ethical Questions in Fish Farming. Journal of Agricultural and Environmental Ethics.

[CR10] Bovenkerk B, Meijboom FLB (2013). Fish welfare in aquaculture: Explicating the chain of interactions between science and ethics. Journal of Agricultural and Environmental Ethics.

[CR11] Bradley B, Višak T, Garner R (2016). Is death bad for a cow?. The ethics of killing animals.

[CR12] Braithwaite V (2010). Do fish feel pain?.

[CR13] Brando, S. 2016. Wild animals in entertainment. In B. Bovenkerk & J. Keulartz (Eds.), Animal ethics in the age of humans; blurring boundaries in human-animal relationships, the international library of environmental, agricultural and food ethics 23: 295-318. Cham, Switzerland: Springer International Publishing.

[CR14] Brando S, Buchanan-Smith HM (2018). The 24/7 approach to promoting optimal welfare for captive wild animals. Behavioural Processes.

[CR15] Broom DM (2011). A history of animal welfare science. Acta Biotheoretica.

[CR16] Bruijnis MRN, Meijboom FLB, Stassen EN, Meijboom FLB, Stassen EN (2016). Premature culling of production animals; ethical questions related to killing animals in food production. The end of animal life: A start for ethical debate; ethical and societal considerations on killing animals.

[CR17] Carroll AB (1979). A three**-**dimensional conceptual model of corporate social performance. Academy of Management Review.

[CR18] Carroll AB (1991). The pyramid of corporate social responsibility: Toward the moral management of organizational stakeholders. Business Horizons.

[CR19] Chandroo KP, Duncan IJH, Moccia RD (2004). Can fish suffer?: Perspectives on sentience, pain, fear and stress. Applied Animal Behaviour Science.

[CR20] Chappell RY (2013). Value Receptacles. Noûs.

[CR21] Clayton NS, Bussey TJ, Dickinson A (2003). Can animals recall the past and plan for the future?. Nature Reviews Neuroscience.

[CR22] Cochrane A (2013). From human rights to sentient rights. Critical Review of International Social and Political Philosophy.

[CR23] Cochrane A, Garner R, O’Sullivan S (2016). Labour rights for animals. The political turn in animal ethics.

[CR24] Crook RJ, Walters ET (2011). Nociceptive behavior and physiology of Molluscs: Animal welfare implications. ILAR Journal.

[CR25] Davis S (2008). What would the world be like without animals for food, fiber, and labor? Are we morally obligated to do without them?. Poultry Science.

[CR26] Dawkins MS (1980). Animal suffering: The science of animal welfare.

[CR27] De Goede D, Gremmen B, Rodenburg TB, Bolhuis JE, Bijma P, Scholten M, Kemp B (2013). Reducing damaging behaviour in robust livestock farming. NJAS – Wageningen Journal of Life Sciences.

[CR28] De Goede DM, Erens J, Kapsomenou E, Peters M, Röcklinsberg H, Sandin P (2013). Large scale insect rearing and animal welfare. The ethics of consumption: The citizen, the market and the law.

[CR29] Deckers J (2016). Animal (De)liberation: Should the consumption of animal products be banned?.

[CR30] DeGrazia D (1996). Taking animals seriously: Mental life and moral status.

[CR31] DeGrazia D, Singer P (2006). On the question of personhood beyond homo sapiens. In Defence of animals: The second wave.

[CR32] DeGrazia D, Beauchamp TL, Frey RG (2011). The ethics of confining animals: From farms to zoos to human homes. The Oxford handbook of animal ethics.

[CR33] Donaldson S, Kymlicka W (2011). Zoopolis: A political theory of animal rights.

[CR34] Donaldson T, Preston LE (1995). The stakeholder theory of the corporation: Concepts, evidence, and implications. The Academy of Management Review.

[CR35] Duncan IJH (2006). The changing concept of animal sentience. Applied Animal Behaviour Science.

[CR36] Elder MP (2014). The fish pain debate: Broadening Humanity's moral horizon. Journal of Animal Ethics.

[CR37] Elwood RW, Barr S, Patterson L (2009). Pain and stress in crustaceans?. Applied Animal Behaviour Science.

[CR38] Engster D (2006). Care ethics and animal welfare. Journal of Social Philosophy.

[CR39] Eriksson D, Svensson G (2016). The process of responsibility, decoupling point, and disengagement of moral and social responsibility in supply chains: Empirical findings and prescriptive thoughts. Journal of Business Ethics.

[CR40] Farm Animal Council (2009). Farm animal welfare in Great Britain: Past, present and future.

[CR41] Fiorito G, Affuso A, Anderson DB, Basil J, Bonnaud L, Botta G, Cole A, D’Angelo L, De Girolamo P, Dennison N, Dickel L, Di Cosmo A, Di Cristo C, Gestal C, Fonseca R, Grasso F, Kristiansen T, Kuba M, Maffucci F, Manciocco A, Mark FC, Melillo D, Osorio D, Palumbo A, Perkins K, Ponte G, Raspa M, Shashar N, Smith J, Smith D, Sykes A, Villanueva R, Tublitz N, Zullo L, Andrews P (2014). Cephalopods in neuroscience: Regulations, research and the 3Rs. Invertebrate Neuroscience.

[CR42] Foer JS (2009). Eating Animals.

[CR43] Francione G, Francione G, Garner R (2010). The abolition of animal exploitation. The animal rights debate: Abolition or regulation?.

[CR44] Fraser D (2008). Toward a global perspective on farm animal welfare. Applied Animal Behaviour Science.

[CR45] Frazer D (1995). Science, values, and animal welfare: Exploring the ‘inextricable connection’. Animal Welfare.

[CR46] Freeman, R.E., & Velamuri, S.R. 2008. A New Approach to CSR: Company Stakeholder Responsibility. Available at SSRN: https://ssrn.com/abstract=1186223.

[CR47] Freeman RE, Harrison JS, Wicks AC, Bidhan LP, De Colle S (2010). Stakeholder theory: The state of the art.

[CR48] Garner R (2013). A theory of justice for animals: Animal rights in a nonideal world.

[CR49] Gjerris M, Gamborg C, Röcklinsberg H, Anthony R (2011). The Price of responsibility: Ethics of animal husbandry in a time of climate change. Journal of Agricultural and Environmental Ethics.

[CR50] Gruen, L. 2017. The moral status of animals. *Stanford encyclopedia of philosophy* (Fall 2018 edition), Edward N. Zalta (ed.), https://plato.stanford.edu/entries/moral-animal/.

[CR51] Haraway D (2008). When species meet.

[CR52] Hart SL, Sharma S (2004). Engaging fringe stakeholders for competitive imagination. Academy of Management Executive.

[CR53] Hearne V (1992). What’s wrong with animal rights: Of hounds, horses and Jeffersonian happiness. Harper’s Magazine.

[CR54] Heath J (2014). Morality, competition, and the firm; the market failures approach to business ethics.

[CR55] Isaacs T (2011). Moral responsibility in collective contexts.

[CR56] Janssens MRE, Kaptein M (2016). The ethical responsibility of companies towards animals: A study of the expressed commitment of the fortune global 200. The Journal of Corporate Citizenship.

[CR57] Janssens MRE, Van Wesel F, Linzey A, Linzey C (2018). Leadership, partnership and championship as drivers for animal ethics the Western food industry. Ethical vegetarianism and veganism.

[CR58] Janssens MRE, Van Wesel F (2019). Connecting parties for change, A qualitative study into communicative drivers for animal welfare in the food industry. Food Ethics.

[CR59] Kagan S, Višak T, Garner R (2016). Singer on killing animals. The ethics of killing animals.

[CR60] Kaiser, M.,Godson, S., Buklijas, T., Guckman, P. Allen, K, Bardsley, A., & Lam, M.E. 2021. Towards post-pandemic sustainable and ethical food systems. Food Ethics 6: 4. 10.1007/s41055-020-00084-3 PMC7826152 33521245

[CR61] Kaldewaij F (2013). The animal in morality, justifying duties to animals in Kantian moral philosophy.

[CR62] Kant, I. 1785/1902/1996. Groundwork for the metaphysics of morals (trans: Gregor, M.). Cambridge, UK: Cambridge University Press.

[CR63] Kant, I. 1963. Lectures on ethics (trans: Infield, L.). New York: Harper and Row.

[CR64] Kaptein M, Wempe J (2002). The balanced company: A theory of corporate integrity.

[CR65] Kasperbauer TJ, Sandøe P, Višak T, Garner R (2016). Killing as a welfare issue. The ethics of killing animals.

[CR66] Keeling L, Tunón H, Olmos Antillón G, Berg C, Jones M, Stuardo L, Swanson J, Wallenbeck A, Winckler C, Blokhuis H (2019). Animal welfare and the United Nations sustainable development goals. Frontiers in Veterinary Science.

[CR67] Keulartz, J. 2016. Towards an animal ethics for the Anthropocene. In B. Bovenkerk B. & J. Keulartz (Eds.), Animal ethics in the age of humans: Blurring boundaries in human-animal relationships (the international library of environmental, agricultural and food ethics 23): 243-264. Cham, Switzerland: Springer international publishing AG.

[CR68] Kolk A, Lenfant F (2012). Business-NGO collaboration in a conflict setting. Business & Society.

[CR69] Kolstad I (2007). Why firms should not always maximize profits. Journal of Business Ethics.

[CR70] Leder D (2012). Old McDonald’s had a farm: The metaphysics of factory farming. Journal of Animal Ethics.

[CR71] Linzey A, Linzey C, Linzey A, Linzey C (2018). Introduction: Oxford: The home of controversy about animals. The ethical case against animal experiments.

[CR72] Marino L (2017). Thinking chickens: A review of cognition, emotion, and behavior in the domestic chicken. Animal Cognition.

[CR73] McMullen S (2015). Is capitalism to blame? Animal lives in the marketplace. Journal of Animal Ethics.

[CR74] Meijer E (2017). Political animal voices.

[CR75] Mepham B, Meijboom FLB, Stassen EN (2016). Morality, morbidity and mortality: An ethical analysis of culling nonhuman animals. The end of animal life: A start for ethical debate; ethical and societal considerations on killing animals.

[CR76] Milligan T, Garner R, O’Sullivan S (2016). Putting pluralism first. The political turn in animal ethics.

[CR77] Mitchell RK, Agle BR, Wood DJ (1997). Toward a theory of stakeholder identification and salience: Defining the principle of who and what really counts. Academy of Management Review.

[CR78] Nasr MAF, Nicol CJ, Murrell JC (2012). Do laying hens with keel bone fractures experience pain?. PLoS One.

[CR79] Nussbaum M (2007). Frontiers of justice: Disability, nationality, species membership.

[CR80] OECD-FAO (2018). OECD-FAO guidance for responsible agricultural supply chains.

[CR81] Ohl F, Van der Staay FJ (2012). Animal welfare: At the interface between science and society. The Veterinary Journal.

[CR82] Pachirat T (2011). Every twelve seconds: Industrialized slaughter and the politics of sight.

[CR83] Pettit P (2007). Responsibility incorporated. Ethics.

[CR84] Regan T (1983). The case for animal rights.

[CR85] Rollin BE (1995). Farm animal welfare: Social, bioethical, and research issues.

[CR86] Rollin BE (2006). Animal Rights & Human Morality.

[CR87] Rollin BE (2011). Putting the horse before Descartes: My Life’s work on behalf of animals.

[CR88] Rollin BE, Meijboom FLB, Stassen EN (2016). Death, telos and euthanasia. The end of animal life: A start for ethical debate; ethical and societal considerations on killing animals.

[CR89] Rollin BE (2016). A new basis for animal ethics: Telos and common sense.

[CR90] Sandøe P, Jensen KK, Wathes C, May S, McCulloch S, Whiting M, Corr S (2011). The idea of animal welfare – Developments and tensions. ICVAE – First international conference on veterinary and animal ethics 2011.

[CR91] Scanlon TM (1998). What we owe to each other.

[CR92] Schmitz F, Garner R, O’Sullivan S (2016). Animal ethics and human institutions. The political turn in animal ethics.

[CR93] SER (2018). IMVO Convenant Voedingsmiddelen.

[CR94] Simmons 2016. Animals, Freedom and the Ethics of Veganism. In B. Bovenkerk B. & J. Keulartz (Eds.), Animal Ethics in the Age of Humans: Blurring Boundaries in Human-animal Relationships: 265-277. The International Library of Environmental, Agricultural and Food Ethics 23, Cham, Switzerland: Springer International Publishing AG.

[CR95] Singer P (1975). Animal liberation.

[CR96] Singer P (1981). Expanding the circle: Ethics, evolution, and moral Progress.

[CR97] Singer P (1993). Practical ethics.

[CR98] Special Eurobarometer. 2015. *Special Eurobarometer 442 Report 2015: Attitudes of Europeans towards Animal Welfare*. http://www.eurogroupforanimals.org/eurobarometer

[CR99] Spruijt BM, Van den Bos R, Pijlman FTA (2001). A concept of welfare based on reward evaluating mechanisms in the brain: Anticipatory behaviour as an indicator for the state of reward systems. Applied Animal Behaviour Science.

[CR100] Stoskopf MK (1994). Pain and analgesia in birds, reptile, amphibians, and fish. Investigative Ophthalmology & Visual Science.

[CR101] Sumner LW (1996). Welfare, Happiness & Ethics.

[CR102] Van Liedekerke L, Dubbink W (2008). Twenty years of European business ethics: Past developments and future concerns. Journal of Business Ethics.

[CR103] Van Marrewijk M (2003). Concepts and definitions of CSR and corporate sustainability: Between agency and communion. Journals of Business Ethics.

[CR104] Vanhonacker F, Verbeke W (2014). Public and consumer policies for higher welfare food products: Challenges and opportunities. Journal of Agricultural and Environmental Ethics.

[CR105] Varner GE (2010). A Harean perspective on humane sustainability. Ethics & the Environment.

[CR106] Varner GE (2012). Personhood, ethics, and animal cognition: Situating animals in Hare’s two-level utilitarianism.

[CR107] Višak T (2011). Killing happy animals, explorations in utilitarian ethics.

[CR108] Višak, T. 2015. Environmental ethics. In N. Wedwan & N. (Eds.), *An integrated approach to environmental management*: 337-361. Hoboken, NJ: Wiley.

[CR109] Višak T, Garner R, Višak T, Garner R (2016). Introduction. The ethics of killing animals.

[CR110] Visser W (2014). CSR 2.0: Transforming corporate sustainability and responsibility.

[CR111] Warren MA (1997). Moral status; obligations to persons and other living things.

[CR112] Webster J (1994). Animal welfare: A cool eye towards Eden.

[CR113] Webster J, Turner J, D’Silva J (2006). Ideals and realities: What do we owe to farm animals?. Animals, ethics and trade: The challenge of animal sentience.

[CR114] Webster J (2013). Animal husbandry regained: The place of farm animals in sustainable agriculture.

[CR115] Wemelsfelder F, Hunter EA, Mendl MT, Lawrence AB (2000). The spontaneous qualitative assessment of behavioural expressions in pigs: First explorations of a novel methodology for integrative animal welfare measurement. Applied Animal Behaviour Science.

[CR116] Yeates JW (2010). Death is a welfare issue. Journal of Agricultural Ethics.

